# Current status of stem cell treatments and innovative approaches for stress urinary incontinence

**DOI:** 10.3389/fmed.2022.1073758

**Published:** 2022-12-02

**Authors:** Mehmet Murat Seval, Kazibe Koyuncu

**Affiliations:** ^1^Department of Obstetrics and Gynecology, Ankara University School of Medicine, Ankara, Turkey; ^2^Department of Obstetrics and Gynecology, Medicana Hospital, Istanbul, Turkey

**Keywords:** stem cell, stress urinary incontinence, urogynecology, urethra, sphincter

## Abstract

Stem cells are capable of self-renewal, differentiation, and the promotion of the release of chemokines and progenitor cells essential for tissue regeneration. Stem cells have the potential to develop into specialized cells if given the right conditions, to self-renew and maintain themselves, to generate a large number of new differentiated cells if injured, and to either generate new tissues or repair existing ones. In the last decade, it has become clear that treating lower urinary tract dysfunction with the patient's own adult stem cells is an effective, root-cause method. Regenerative medicine is predicated on the idea that a damaged rhabdosphincter can be repaired, leading to enhanced blood flow and improved function of the sphincter's exterior (striated) and internal (smooth) muscles. Stem cell therapy has the potential to cure stress urinary incontinence according to preclinical models. In contrast, stem cell treatment has not been licensed for routine clinical usage. This article reviews the current state of stem cell for stres urinary incontinence research and recommends future avenues to facilitate practical uses of this potential therapy modality.

## Introduction

It has been estimated that up to half of all adult women experience urinary incontinence at a certain point according to a recent research (1). Ten to 20% of all females are diagnosed with this disorder, and as many as 77% of women in nursing homes suffer from it (2–8). Data from primary care settings show that 37.5% of women between the ages of 30 and 50 suffer from stress incontinence (9). Urinary incontinence was predicted to be the primary diagnosis or chief complaint for 6.8 million women, according to the National Ambulatory and Hospital Medical Care Survey for 2009–2010; 15.3% of these women received treatment in a primary care setting (10). Incontinence is still underdiagnosed and neglected despite how common it is. Less than half of the affected women who seek medical attention—only 25%—are treated (11). It was discovered that untreated incontinence was linked to fractures, trouble sleeping, depression, and UTIs (12–14).

Urinary incontinence has found to affect up to 50% of adult women (1). This disorder affects 10–20% of all women and up to 77% of older women (2–8). Recent findings reveal that almost one third of young women in primary care have stress incontinence (9). It has been estimated 6.8 million women had urine incontinence as their primary diagnosis or chief complaint; 15.3% were treated in a practitioner (10). Incontinence continues to be underdiagnosed and untreated while it has been a very common problem. Only a quarter of affected women shown to seek medical attention, and of those, less than half are treated (11). Untreated incontinence was found to be related to fractures, sleep difficulties, depression, and urinary tract infections (12–14).

The management of adult female urine incontinence is an evolving practice. For some affected women, urine incontinence is bothersome and intrusive enough to justify therapy consideration. Options range from lifestyle modifications to more intrusive surgical procedures (15). Urinary incontinence may be treated with pelvic floor therapy, lifestyle modifications (including fluid optimization), pharmaceutical treatment, or surgery in women who are overall healthy. Instead, women with other major health concerns may view their urine incontinence as a chronic condition, with a focus on symptom reduction rather than complete remission.

Stem cells are self-renewing and capable of differentiating into progenitor cells to replace aged cells suffering apoptosis (16, 17). Existing urine incontinence treatments may be unsatisfactory regardless of the underlying reason, resulting in a considerable decrease in patients' quality of life. So, stem cell research has been risen to the forefront of regenerative medicine (18–20).

Although the studies comprised a very small number of patients, it is possible to consider stem cell injection safe, at least in the short term, because only mild adverse effects were observed. However, there is a great deal of variation in the effectiveness findings between research. Studies using adipose-derived stem cells showed only a slight or no benefit when looking at subjective or objective outcomes. Muscle-derived stem cells and human cord blood stem cells were found to have greater benefits in terms of patient satisfaction. In terms of the instrumental results, there was too much variation between trials to make any solid claims. In addition to using different cell lines, variations in sample size, cell injection volume, and follow-up period all contribute to non-comparable findings among research.

Diverse preclinical models were developed to test the therapeutic effects of stem cells for stress urinary incontinence, but clinical studies in human are scarce. This review examines the present and future directions of stem cell treatment research for stress urinary incontinence.

## Stress urinary incontinence pathophysiology

Stress urinary incontinence (SUI) is caused by a variety of factors and typically be attributed to mechanical and functional factors. Myogenic, connective tissue, and hormonal alterations are significant variables. In addition, muscle cell density falls because of natural aging process and decreased muscle function in rhabdosphincter, with the overall volume reducing from 88% at birth to 34% in the 90th year of life (21). Female SUI is frequently caused by multiple factors, including dysfunctions of the sphincter and nerve injury. The mid-urethral sling has the benefit of requiring less intervention time. The rate of any re-operation was 5.5–6.9% in long term follow-up (22). However, several organizations has frequently issued warnings against the use of mesh materials in the treatment of female urinary incontinence as the result of many severe adverse events (23).

Preclinical studies have progressively used several stem cell types to treat SUI in recent years. Determine the best cell type for therapeutic usage by carefully weighing the benefits and drawbacks of each sort. The idea of regenerative medicine is based on the rehabilitation of a dysfunctional rhabdosphincter, with enhancements in the activity of the sphincter's (external and/or internal) muscles as well as its blood flow.

## Stem cell types

Because a human embryo at the blastocyst stage can be viewed as an individual human, it is ethically unacceptable to isolate embryonic stem cells. Stem cells can be taken from bone marrow, muscle tissue, adipose tissue (24–28), and testicular tissue, among other sources (29). Adult stem cells could be derived from multiple sources like adipose tissue, bone marrow or muscle tissue etc. may provide a good option for regenerative therapy since they have a minimal chance of developing into cancer, may be transferred autologously without rejection risk, and ethical debate. The atrophied, damaged musculature is brought back to normal function by promoting muscle and nerve regeneration by injecting adult stem cells into the wounded rhabdosphincter. Stem cells regenerate the matrix and the muscle cells that maintain normal contraction function and continence. This action is made possible by the cells' prior development into neurones or striated muscle cells, which may repair damaged parts.

Muscle-derived stem cells (MDSCs) and adipose tissue-derived stem cells (ADSCs) have been examined more extensively than other cell types to date, however studies on urine-derived stem cells, bone marrow-derived stem cells, amniotic fluid-derived stemcells, and umbilical cord blood stem cells are increasing. Autologous cells that may be obtained with few invasive procedures, in high quantities, and for use in stem cell therapies are the ideal characteristics.

## Methods

We looked at the outcome in terms of UI reduction and continence restoration following treatment. PRISMA standards were used to guide the literature search that was conducted. Only data obtained from clinical studies involving humans, in female patients with SUI, were eligible for inclusion in the study. No institutional review board permission was needed. The literature stem cell therapy for stress urinary incontinence patients published up through November 2022 was combed using four internet databases (PubMed, Cochrane Library and Scopus). The search method was modified for each database, but the overarching keywords were (Stress urinary incontinence) and (Regenerative medicine OR Cell- and Tissue-Based Therapy OR Stem Cell Transplantation OR Stem cell).

## Clinical trials with stem cells for stress urinary incontinence

In stem cell clinical trials, SUI has received the most attention (30–44). Different types of stem cells have been shown to be therapeutically effective and safe when used to treat SUI in the literature (45). ADSCs are now the most prevalent kind of stem cell utilized in plastic transplantation. A large quantity of adipose tissue is retrievable after liposuction, and repeated sampling is possible. ADSCs differentiate *via* adipogenesis, osteogenesis, chondrogenesis, and myogenesis (43, 46). Kuismanen et al. reported that after autologous ADSC injections into the human urethra, a cough test was negative for all patients. The total UI ratings increased considerably (40). This study also demonstrated that the use of stem cells to treat SUI is safe and tolerated; nevertheless, more research is required. Arjmand et al. enrolled 10 female patients with SUI and demonstrated a substantial improvement at 2–24 weeks following ADSC injection (47). Kuismanen et al. enrolled five female SUI patients treated with ADSCs. Three patients passed the cough test at the 1-year follow-up, whereas the other two did not. The surgery has a 60% success rate (40). The majority of prior research was limited by small sample size and gathering of primarily short-term outcomes. Success rates varied between 30 and 100%.

MDSCs have been widely researched as a means of SUI therapy (48). Muscle biopsies taken under local anesthesia may result in low morbidity (49) when MDSCs are cultured. The extracted muscle tissue must be enlarged *in vitro* and then reinjected into the paraurethral area (50). MDSCs have been proven to have a high regenerative capacity (32). MDSCs can be administered transurethrally or periurethrally into the rhabdosphincter to enhance sphincter function and as blocking agents (48, 50). After unsuccessfully trying several treatments for SUI, Carr et al. injected autologous, MDSCs into the urethral sphincters of eight women. Six women improved in pad tests, urination diaries, and quality of life questionnaires after a year, and one of these women reached perfect continence (32). Thirty-eight patients and a range of stem cell doses were added to the trial in 2013. Compared to those given lower dosages, patients treated with larger doses saw greater symptom relief (33). After myoblast and fibroblast injection, 123 female patients by Mitterberger et al. shown a significant improvement in SUI (follow-up at 62.9 months), 79% (*n* = 94) of the patients were stable at the 1-year check-up, while 13% (*n* = 16) showed significant improvement (41). Stangel-Wojcikiewicz et al. enrolled 16 women and noted an improvement in 25% of women based on clinical and urodynamic outcomes (36). Sharifiaghdas et al. conducted a prospective cohort research involving 10 women receiving MDSCs for the treatment of SUI (51). Three patients regained full continence after 3 years of follow-up, assesed with a cough stress test, a 1-h pad test, and questionnaires. Three patients did not respond to the medication, whereas four individuals shown great improvement. Gerullis et al. included 222 patients who had had a urological procedure and were given autologous MDSCs (52). After a 6- to 12-month follow-up, 12% of patients were continent, 42% improved, and 46% had chronic urinary incontinence. In another study 123 women with SUI were recruited treated with MDSC injections (53). At the 1-year follow-up, 79% of the women were totally continent, whereas 13 and 8% improved significantly. In another study, Mitterberger et al. selected 20 women with SUI and gave them 1–3 107 MDSCs (41). At the 1-year follow-up, 18 patients had been cured, and the SUI of two patients had improved. The therapeutic effect was constant during a 2-year follow-up and quality of life scores significantly improved. In another study, 38 patients with SUI were treated with MDSCs (53). The improvement in SUI was examined using objective outcomes and patient and clinician perceptions after a 2-year follow-up, and all indicated a substantial improvement. They found that MDSC injection is possible and safe in patients with SUI, and that the patients' quality of life improved dramatically. Sebe et al. enrolled 12 female SUI patients and treated them with MDSCs (30). At 12 months, three of the twelve patients (25%) were dry on the pad test, while seven (58.3%) of the other patients improved. Six of the twelve (50%) patients reported an improvement in their quality of life.

Umbilical cord blood stem cells (USCs) may be extracted from human umbilical cords (54). USCs are regarded to be more capable of differentiating than adult stem cells (54). The collection of USCs does not entail any intrusive procedures, which is an additional benefit. Additionally, there is a low risk of graft-vs.-host disease and virus contamination with USCs. Matching HLA types might be less rigorous. In addition, USCs are available *via* donor-based banking systems (55). Lee et al. recruited and implanted USCs into 39 women with urinary incontinence (55). The submucosal area of the proximal urethra was injected with USCs with 4.3 ± 1.9 × 108 cells per 2 mL of media at the 4 and 8 o'clock positions. At the 1-year follow-up, 36% of patients were completely continent, and 36% had markedly improved urinary incontinence. Nonetheless, 27% of patients did not improve.

## Conclusion

Although the clinical research on stem cell therapy for the treatment of SUI reveal promising outcomes with significant promise, these short-term results must be viewed with caution. The outcomes of numerous clinical trials are debatable. The endurance of stem cells is a challenge. Rapid reabsorption of body fat ensues. Suction damages cell membranes, and only 10–30% of fat cells are detectable 6 months after application (56). In all documented urinary incontinence clinical studies, autologous stem cells were injected transurethrally, periurethrally or transperineal. The results of various injection methods have been rarely compared (57–59). A recent Cochrane review of studies comparing urethral injection for the treatment of female SUI found no evidence for a significantly better application type (60). Jaeger et al. reported a unique methodology for delivering MSCs into the external urethral sphincter that utilized a method without needle using waterjet technology (61). The number of transplanted cells varied considerably. The range of injected cells was between 1.8 × 10^6^ and 50 × 10^6^ cells. The greatest number of cells was injected while using MDSCs (62). In each study, the volume of injected cells was <10 ml. Due to a lack of clarity surrounding stem cell-based therapy, different cell dosages are utilized. However, it is undeniable that the concept of regenerative medicine leads to regeneration of the injured rhabdosphincter, as well as an improvement in the function of the external and internal sphincters and the blood supply of sphincter muscle. Stem cell therapies have become appealing tools as they are biocompatible and not causing adverse inflammatory reactitons. However, the existing data are quite varied, making comparisons between cell types or cell coating processes problematic. In addition, few clinically relevant animal models have been utilized, resulting in inconsistent findings. Lastly, a thorough evaluation of the basic mechanisms of stem cells linked with the host reaction is essential.

## Author contributions

MS and KK have made substantial contributions to the conception or design of the work and drafting the work or revising it critically for important intellectual content. All authors provide approval for publication of the content and agreed to be accountable for all aspects of the work in ensuring that questions related to the accuracy or integrity of any part of the work are appropriately investigated and resolved.

## Conflict of interest

The authors declare that the research was conducted in the absence of any commercial or financial relationships that could be construed as a potential conflict of interest.

## Publisher's note

All claims expressed in this article are solely those of the authors and do not necessarily represent those of their affiliated organizations, or those of the publisher, the editors and the reviewers. Any product that may be evaluated in this article, or claim that may be made by its manufacturer, is not guaranteed or endorsed by the publisher.
